# Evaluation of Processing Technology for *Triarrhena sacchariflora* (Maxim.) Nakai for Ethanol Production

**DOI:** 10.1371/journal.pone.0114399

**Published:** 2014-12-09

**Authors:** Fengqin Gao, Fuyu Yang, He Zhou, Qizhong Sun, Yunwei Zhang, Michael A. Brown

**Affiliations:** 1 Department of Grassland Science, College of Animal Science and Technology, China Agricultural University, Beijing 100193, PR China; 2 Grassland Research Institute, Chinese Academy of Agricultural Sciences, Hohhot 010010, PR China; 3 B&B Research & Development, 16835 SW 27th St., El Reno, OK 73036, United States of America; Glasgow University, United Kingdom

## Abstract

The effects of dilute H_2_SO_4_ concentration, forage:sulfuric acid ratio, digestion time, and digestion temperature were evaluated to determine effects on ethanol yield of *Triarrhena sacchariflora* (Maxim.) Nakai. Twenty single factor experiments were conducted to evaluate H_2_SO_4_ concentration (0.5, 1.0, 1.5, 2.0, and 2.5%, w/w), forage:sulfuric acid ratio (1∶6, 1∶8, 1∶10, 1∶12, and 1∶14, g/ml), digestion time (15, 30, 45, 60, and 90, min), digestion temperature (80, 100, 110, 120, and 125 °C) for 3 replicates of the 5 levels of each factor. Based on results of the single factor experiments, an incomplete factorial was designed to evaluate ethanol yield from the best combinations of single factors. Finally, the best combination was tested by enzymatic hydrolysis and fermentation experiment in selected combinations according to pretreatment results. Percentage cellulose, hemicellulose, and lignin contents of forage residue after pretreatment, and glucose and xylose concentrations of the filtrate were analyzed prior to enzymatic hydrolysis, and percentage crystallinity was observed in untreated grass and pretreated residue. In addition, the solid residues were then hydrolysed and fermented by cellulase and yeast, the concentrations of glucose and ethanol being monitored for 96 h. Results showed that the order of the effect of main effect factors was as follows: digestion temperature > dilute H_2_SO_4_ concentration > digestion time > forage:sulfuric acid ratio. The best process parameters evaluated were sulfuric acid concentration of 1.5%, forage:sulfuric acid ratio of 1∶6, digestion time of 15 min, and digestion temperature of 120°C. With this combination of factors, 80% of the cellulose was hydrolysed in 96 h, and 78% converted to ethanol. The findings identified that hemicelluloses were the key deconstruction barrier for pretreatment of *Triarrhena sacchariflora* (Maxim.) Nakai for ethanol production. The results of this research provide evidence of appropriate combinations of processing factors for production of ethanol from *Triarrhena sacchariflora* (Maxim.) Nakai.

## Introduction

Fossil energy is a finite, non-renewable resource that has significant impact on the world economy. Current world-wide, energy consumption is around 130×10^8^ t coal equivalent per year, of which fossil energy comprises over 80% [1]. Energy demand and consumption is expected to increase as a function of both world economy and population growth. Spot shortages of fossil energy commonly occur and the increased utilization of fossil fuels suggests it is reasonable to predict future depletion of fossil energy throughout the world. Moreover, utilization of fossil energy results in SO_2_, CO, and CO_2_ emissions, which are believed to contribute to climate change. The potential for depletion of fossil fuels combined with the environmental impacts associated with use of these fuels has resulted in considerable effort to develop technology to produce energy from renewable resources that has a lesser environmental impact. Ethanol can substitute for gasoline in a fuel mix of ethanol and gasoline and can be produced from renewable resources. Ethanol can be produced from grains such as corn, but there is some indication that the energy required for ethanol production from grain exceeds the energy yield in the ethanol product [2], [3]. Additionally, use of grains for fuel ethanol production diverts those grains from human and livestock consumption and results in increased grain and food prices. A more reasonable alternative for ethanol production is fermentation of grasses and crop aftermath. Carbohydrates in forages are more complex than grains and the chemical bonds in the cellulose, hemicellulose, and lignin of forages must be broken to reduce the carbohydrates to simpler and fermentable fractions. Cellulosic ethanol production has good potential for renewable and clean energy production. However, different strategies will need to be adopted to identify appropriate grasses for ethanol production or when designing processing conditions to efficiently convert a specific biomass feedstock into sugars [4]. So, considerable work remains to determine optimal biomass sources as well as optimal processing methods.

*Triarrhena sacchariflora* (Maxim.) Nakai (Amur Silvergrass) is a grass of the *Miscanthus* spp. of Gramineae, abundant in the grasslands of northern China, and has potential for utilization in ethanol production. Plant height averages 3–4 m and annual dry matter yield averages 22.5–30 t/ha [5]. *Triarrhena sacchariflora* (Maxim.) Nakai has extensive adaptability, strong regeneration capacity, high caloric value of dry matter, and strong potential for carbon sequestration. Consequently, it has excellent potential for cellulosic ethanol production. However, there exists a need for further study on optimal techniques for digestion of this forage.

Cellulosic biomass is considered one of the most promising materials for producing fuel ethanol due to its global availability and environmental benefits. However, lignocellulose biomass must be pretreated [6] to reduce the complex carbohydrates to more simple carbohydrates to improve the conversion to ethanol during the fermentation process [7]. Acid, alkaline, mechanical processing, steam pretreatment, liquid hot water and ammonia pretreatment processes have been extensively investigated [8]–[13]. Among all the pretreatment methods, dilute acid pretreatment has been shown to be most effective and is most widely used in commercial production [14]–[17]. However, there is a need to determine optimal combinations of sulfuric acid concentration, forage:sulfuric acid ratio, digestion time, and digestion temperature with reference to *Triarrhena sacchariflora* (Maxim.) Nakai.

Thus, the objectives of this study were to evaluate the efficacy of combinations of sulfuric acid concentrations, forage:sulfuric acid ratios, digestion times, and digestion temperatures for pretreatment, and investigate the key deconstruction barrier of dilute H_2_SO_4_ pretreatment of *Triarrhena sacchariflora* (Maxim.) Nakai for ethanol production.

## Materials and Methods

### Materials

*Triarrhena sacchariflora* (Maxim.) Nakai was gathered randomly from 2 m^2^ plots in Xiao tang shan grassland of the Beijing Agro-forestry Academy from 40°17′N to 116°39′E on Oct.8^th^ 2011. Samples were sun-cured in the field, cut into 2–4 cm lengths, and then stored in sealed plastic bags at 4°C. Prior to initiation of the experiment, dry matter was determined using a drier oven (DHG-9240A, Jinghong Lab Instrument Co. Ltd, Shanghai, China) at 105°C to constant moisture. Chemical compositions of *Triarrhena sacchariflora* (Maxim.) Nakai are shown in Table 1.

**Table 1:** Chemical compositions of *Triarrhena sacchariflora* (Maxim.) Nakai.

Item	*Triarrhena sacchariflora* (Maxim.) Nakai (%)
Cellulose	50.63
Hemicellulose	28.49
Lignin	9.41
Acid detergent fiber	61.13
Neutral detergent fiber	89.62
Crude protein	8.03
Crude ash	1.09
Crystallinity	5.85

### Methods

#### Single factor experiment of sulfuric acid pretreatment

Single factor experiments were designed as one-way classifications with 3 replicates to evaluate: 1) different levels of sulfuric acid concentration holding forage:sulfuric acid ratio, digestion time, and digestion temperature constant; 2) different levels of forage:sulfuric acid ratio holding sulfuric acid concentration, digestion time, and digestion temperature constant; 3) different levels of digestion time holding sulfuric acid concentration, forage:sulfuric acid ratio, and digestion temperature constant; and 4) different levels of digestion temperature holding sulfuric acid concentration, forage:sulfuric acid ratio, and digestion time constant (Table 2).

**Table 2:** Single factor experimental design of *Triarrhena sacchariflora* (Maxim.) Nakai with dilute sulfuric acid pretreatment.

Influence factors	Sulfuric acid concentration (%, w/w)	Forage:sulfuric acid ratio (g/ml)	Digestion time (min)	Digestion temperature (°C)
Sulfuric acid concentration	0.5	1∶8	30	120
	1.0	1∶8	30	120
	1.5	1∶8	30	120
	2.0	1∶8	30	120
	2.5	1∶8	30	120
Forage:sulfuric acid ratio	1.5	1∶6	30	120
	1.5	1∶8	30	120
	1.5	1∶10	30	120
	1.5	1∶12	30	120
	1.5	1∶14	30	120
Digestion time	1.5	1∶8	15	120
	1.5	1∶8	30	120
	1.5	1∶8	45	120
	1.5	1∶8	60	120
	1.5	1∶8	90	120
Digestion temperature	1.5	1∶8	30	80
	1.5	1∶8	30	100
	1.5	1∶8	30	110
	1.5	1∶8	30	120
	1.5	1∶8	30	125

Dried *Triarrhena sacchariflora* (Maxim.) Nakai (10 g) and corresponding sulfuric acid solution were put into 500 ml triangular flasks (Beibo, Sichuan, China), sealed using aluminium-foil paper, and transferred to a sterilizer (LDZX-75KBS, Shenan Medical Instrument Co. Ltd, Shanghai, China). After pretreatment, the sample was filtered by four layers of gauze, the residue was washed by tap water until neutral, dried at 40–60°C, and filtrate was stored in 50 ml centrifuge tubes at −20°C for glucose and xylose analysis.

#### L_8_ (2^4^) incomplete factorial experiment of sulfuric acid pretreatment

Based on the results of the single factor experiments, the two most efficacious factors were selected to include in an incomplete factorial treatment design. The factors selected were as follows: sulfuric acid concentrations (1.0 and 1.5%, w/w), forage:sulfuric acid ratios (1∶6 and 1∶8, g/ml), digestion times (15 and 30, min), and digestion temperatures (110 and 120 °C). Treatment combinations used in the incomplete factorial are shown in Table 3.

**Table 3:** Incomplete factorial experimental design of *Triarrhena sacchariflora* (Maxim.) Nakai with dilute sulfuric acid pretreatment.

Factors	A Sulfuric acid concentration (%, w/w)	B Forage: sulfuric acid ratio (g/ml)	C Digestion time (min)	D Digestion temperatures (°C)
Experiment No.				
1	1 (1.0)	1 (1∶6)	1 (15)	1 (110)
2	1	1	1	2 (120)
3	1	2 (1∶8)	2 (30)	1
4	1	2	2	2
5	2 (1.5)	1	2	1
6	2	1	2	2
7	2	2	1	1
8	2	2	1	2

#### Experiment of enzymatic hydrolysis and fermentation

Pretreatments for treatment combinations of sulfuric acid concentrations, forage:sulfuric acid ratios, digestion times, and digestion temperatures were as follows: 1) untreated control; 2) 1.5%, 1∶6, 15 min, 110°C; 3) 1.5%, 1∶8, 30 min, 120°C; 4) 1.5%, 1∶6, 15 min, 120°C. After pretreatments according to above treatment combinations, three replicates of 10 g residue samples were cut into 1–2 cm lengths and were put into micro-fermentation tanks (100 ml) and then a solution of 3% peptone as nutrition for yeast was added to the micro-fermentation tanks at 10% of total solution in system onset of the fermentation and the whole micro-fermentation tanks was sterilized at 121°C for 20 min. These micro-fermentation tanks were removed to superclean bench until solution temperature decreased to 30°C, thermal tolerant alcohol active dry yeast (Angel Yeast Co. Ltd, Chifeng, China) was added to the solution at a concentration of 0.03% (w/v) and a commercial cellulase with CMCase activity of 27000 U/g made from *Acremonium cellulolyticus* (Meiji Seika Kaisha Ltd, Tokyo, Japan) was added at 20 FPU/g DM (dry solid matter). Enzymatic saccharification was performed in citric acid-sodium citrate buffer of 4.8 pH at 34°C with shaking at 100 r/min for 96 h using simultaneous saccharification and fermentation (SSF) process. During the fermentation, the glucose and ethanol concentrations were determined.

#### Analytical methods

The cellulose, hemicellulose and lignin contents of pre- and post-treatment were determined by method described by Van Soest [18] employing ANKOM A2000i analysis methods (A2000i, Ankom Technology Co., Fairport, New York, US). Glucose and xylose were analyzed by High Performance Liquid Chromatography (LC1200-HPLC, Agilent Technologies Co. Ltd, Santa Clara, California, US). The analytical conditions were as follows: Agilent carbohydrate column at 30°C, methyl cyanide­water ratio of 80∶20, refractive index detector at 25°C with 40 MPa in velocity of flow of 1 ml/min, sample size of 10 ul. The ethanol concentration was measured with the HP 6890 Series GC system (GC-2014, Shimadzu Co., Japan) and the PEG-20M column under the conditions according to the literature [19]. Crystallinity was determined by X-ray diffraction (X'Pert Pro MPD, PANalytical Co., Almelo, Netherlands), and crystallinity of *Triarrhena sacchariflora* (Maxim.) Nakai is reported relative to the 50% crystallinity of SiO_2_.

#### Statistical

Experimental data from single factor experiments were analyzed as one-way classifications using PROC MIXED (SAS, Cary, NC, USA). Generally treatments were replicated 3 times but missing data occurred for some replications and treatments. Incomplete factorial experiments were analyzed using extreme difference and general equilibrium theory [20]. Linear contrast among least squares means were done using Tukey's LSD at P<0.05.

## Results

### Single factor experiment of sulfuric acid pretreatment

#### Effect of sulfuric acid pretreatment on dry matter digestion

**Figure 1:**
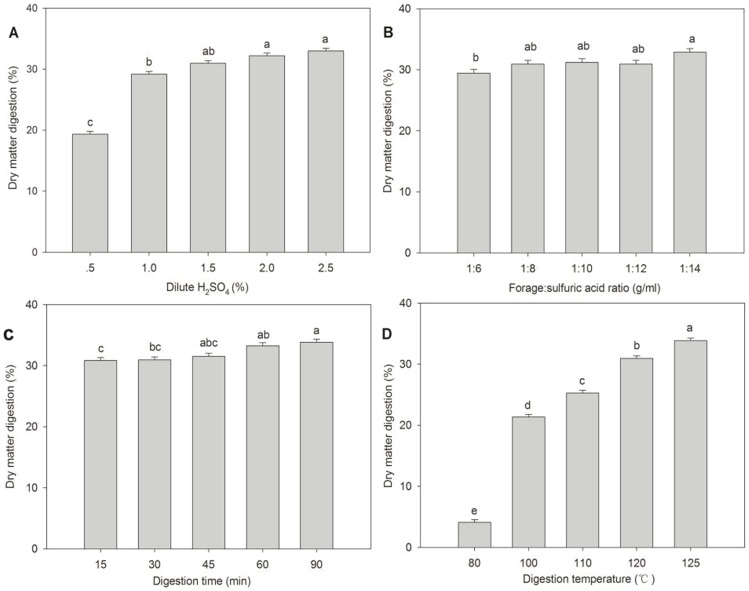
Dry matter digestion treated with various dilute H_2_SO_4_ pretreatments: (A) different H_2_SO_4_ concentrations with fixed forage:sulfuric acid ratio (1∶8), fixed digestion time (30 min), and fixed digestion temperature (120°C); (B) different forage:sulfuric acid ratios with fixed H_2_SO_4_ concentration (1.5%), fixed digestion time (30 min), and fixed digestion temperature (120°C), (C) different digestion times with fixed H_2_SO_4_ concentration (1.5%), fix forage:sulfuric acid ration (1∶8), and fixed digestion temperature (120°C); and (D) different digestion temperatures with fixed H_2_SO_4_ concentration (1.5%), fixed forage:sulfuric acid ratio (1∶8), and fixed digestion time (30 min) for *Triarrhena sacchariflora* (Maxim.) Nakai. Analyses were performed in triplicate with the error bars representing the corresponding standard errors.

Percentage dry matter digestion for samples that were pretreated with sulfuric acid for four single factors are shown in Fig. 1. Generally, dry matter digestion all tended to increase among four single factor conditions, but the highest value was not difference (P>0.05).

Dry matter digestion increased with increased concentration of sulfuric acid (Fig. 1A). When sulfuric acid concentration was 0.5%, dry matter digestion was significantly lower than the other groups (P<0.01), and sulfuric acid concentration of 1.0% had lower dry matter digestion than concentrations of 2% and 2.5%. Increasing sulfuric acid concentration could promote dry matter digestion, but dry matter digestion decreased with increasing sulfuric acid concentration. The effect of forage:sulfuric acid ratio on dry matter digestion is shown in Fig. 1B. A forage:sulfuric acid ratio of 1∶14 had a higher dry matter digestion than a 1∶6 ratio (P<0.05), but there was little evidence of other effects on forage:sulfuric acid ratio on dry matter digestion. Dry matter digestion slowly increased with increased digestion time (Fig. 1C). A digestion time of 90 min resulted in greater dry matter digestion than 15 or 30 min (P<0.05) and a digestion time of 60 min resulted in greater dry matter digestion than 15 min (P<0.05). Dry matter digestion for least and greatest digestion times (15 min and 90 min) were 30.83%, and 33.83%, respectively, suggesting that prolonging digestion time did not result in appreciably larger dry matter digestibility. The effect of digestion temperature on dry matter digestion is shown in Fig. 1D. Digestion temperature had the greatest effect on dry matter digestion, which increased at each temperature from 80°C to 125°C (P<0.01). The dry matter digestion at 100°C increased by 5 times of that at 80°C, which indicated that dry matter digestion was most efficacious at temperature equal or greater than 100°C. However, the increase in dry matter digestion appeared to decrease after 100°C. The reason may be that generated steam pressure destroys the structure of lignocellulose at 100°C pretreatment, and further temperature increases did not appreciably further deconstruct the lignocelluloses complex. This result is similar to other studies evaluating the effects of steam on lignocellulose material [21]–[23]. Results evaluating the effects of different levels of sulfuric acid concentrations, forage:sulfuric acid ratios, digestion times, and digestion temperatures on lignocelluloses digestion suggest that increases in digestion temperatures to approximately 100°C was more efficacious than changes in sulfuric acid concentration, forage:sulfuric acid ratio, or digestion time.

#### Effect of single factor pretreatment on lignocellulosic components and crystallinity

Effect of single factor pretreatments on lignocellulosic components and crystallinity is shown in Table 4–7. Analysis of the grass residues showed that the percentage cellulose hydrolysis was very low, and percentage hemicellulose hydrolysis was very high, while percentage lignin removal was more variable. Lignin removal had no evident effect on cellulose and hemicellulose hydrolysis in dilute sulfuric acid pretreatment for *Triarrhena sacchariflora* (Maxim.) Nakai. However, there was a certain proportional relation between cellulose and hemicellulose hydrolysis. In our paper, the results suggested that hemicellulose likely played an important role in higher levels of hemicellulose for *Triarrhena sacchariflora* (Maxim.) Nakai, similar to other research results [4].

**Table 4:** Means and standard errors for lignocellulosic components and means for crystallinity for different sulfuric acid concentrations, N = 3^1^.

Experiment treatment	Factor level	Cellulose hydrolysis (%)	Hemicellulose hydrolysis (%)	Lignin removal (%)	Relative crystallinity (%)
Sulfuric acid concentration (%, w/w)	0.5	9.48±0.72^c^	43.21±1.23^d^	19.27±1.39^a^	5.70
	1.0	14.33±0.72^b^	72.14±1.23^c^	18.62±1.13^a^	5.76
	1.5	15.95±0.72^ab^	78.84±1.23^b^	10.74±1.39^b^	5.88
	2.0	15.35±0.72^ab^	86.10±1.23^a^	6.13±1.39^b^	6.76
	2.5	18.05±0.72^a^	82.72±1.23^ab^	4.92±1.13^b^	6.78

1 Means with larger standard errors in a column are based on a sample size of N = 2.

abcd Means within a column with differing superscripts differ (P<0.05).

**Table 5:** Means and standard errors for lignocellulosic components and means for crystallinity for different forage:sulfuric acid ratios, N = 3^1^.

Experiment treatment	Factor level	Cellulose hydrolysis (%)	Hemicellulose hydrolysis (%)	Lignin removal (%)	Relative crystallinity (%)
Forage:sulfuric acid ratio (g/ml)	1∶6	7.06±0.66^bc^	80.29±1.60^a^	4.99±1.23^a^	5.80
	1∶8	11.54±0.66^a^	78.85±1.60^a^	10.59±1.23^a^	5.88
	1∶10	9.22±0.66abc	84.14±1.60^a^	6.96±1.00^a^	5.85
	1∶12	6.37±0.66^c^	84.70±1.60^a^	9.82±1.00^a^	5.86
	1∶14	9.95±0.66^ab^	85.55±1.60^a^	10.33±1.00^a^	5.85

1 Means with larger standard errors in a column are based on a sample size of N = 2.

abc Means within a column with differing superscripts differ (P<0.05).

**Table 6:** Means and standard errors for lignocellulosic components and means for crystallinity for different digestion times, N = 3^1^.

Experiment treatment	Factor level	Cellulose hydrolysis (%)	Hemicellulose hydrolysis (%)	Lignin removal (%)	Relative crystallinity (%)
Digestion time (min)	15	9.10±0.52^b^	77.08±1.40^c^	13.91±0.88^a^	3.99
	30	11.54±0.52^a^	78.85±1.40^bc^	10.59±1.08^ab^	4.88
	45	7.36±0.52^b^	84.02±1.40^ab^	6.50±1.08^b^	6.51
	60	9.69±0.52^ab^	86.68±1.40^a^	7.06±0.88^b^	6.12
	90	9.76±0.52^ab^	89.30±1.40^a^	0.88±1.08^c^	6.29

1 Means with larger standard errors in a column are based on a sample size of N = 2.

abcd Means within a column with differing superscripts differ (P<0.05).

**Table 7:** Means and standards errors for lignocellulosic components and means for crystallinity for different digestion temperatures, N = 3^1^.

Experiment treatment	Factor level	Cellulose hydrolysis (%)	Hemicellulose hydrolysis (%)	Lignin removal (%)	Relative crystallinity (%)
Digestion temperature (°C)	80	2.07±0.61^d^	8.80±1.02^e^	18.44±1.33^a^	4.76
	100	4.61±0.61^d^	56.51±1.24^d^	10.73±1.63^b^	5.16
	110	8.77±0.50^c^	69.97±1.02^c^	13.64±1.63^ab^	5.58
	120	11.54±0.50^b^	78.85±1.02^b^	10.59±1.63^b^	6.01
	125	14.54±0.50^a^	84.71±1.02^a^	12.14±1.33^ab^	6.88

1 Means with larger standard errors in a column are based on a sample size of N = 2.

abcde Means within a column with differing superscripts differ (P<0.05).

Cellulose hydrolysis generally increased with increased sulfuric acid concentration (Table 4); 0.5% sulfuric acid was lesser than the other concentrations (P<0.05), while 1.0%, 1.5% and 2.0% concentrations of sulfuric acid were similar (P>0.05). Hemicellulose hydrolysis also increased with increased sulfuric acid concentration; but at a lesser percentage with increasing sulfuric acid concentration. Lignin removal had an inverse relation with cellulose and hemicellulose hydrolysis in sulfuric acid pretreatment. Lignin removal decreased with increased sulfuric acid concentration; lignin removal at 0.5% and 1.0% were similar (P>0.05), and higher than other sulfuric acid concentrations (P<0.05). This suggests that high or low lignin removal was not a major impediment to cellulose hydrolysis of *Triarrhena sacchariflora* (Maxim.) Nakai. Crystallinity generally increased with increasing sulfuric acid concentration, which generally has an inverse relation with lignin removal.

Effect of forage:sulfuric acid ratio on lignocellulosic components and crystallinity is shown in Table 5. Change of forage:sulfuric acid ratio had little effect on hemicellulose hydrolysis, lignin removal, or crystallinity (P>0.05). This suggests that, generally, increases or decreases in forage:sulfuric acid ratio are not a major factor in change crystallinity of crystalline region or lignocellulose deconstruction. However, there was some indication that changes in the forage:sulfuric acid ratio had some influence on cellulose hydrolysis. A ratio of 1∶8 was 11.54% and higher than 1∶6 and 1∶12 (P<0.05).

Effect of digestion time on lignocellulosic components and crystallinity is shown in Table 6. Cellulose hydrolysis varied a little, hemicellulose hydrolysis increased, lignin removal decreased, and the crystallinity increased with increasing digestion time. Cellulose hydrolysis at 30 min was the highest (11.54%), and higher than 15 and 45 min digestion time (P<0.05), which indicated that the best time of cellulose hydrolysis is probably around 30 min. Hemicellulose hydrolysis increased with increasing digestion time with hydrolysis of hemicelluloses at 60 and 90 min exceeding those at 15 and 30 min (P<0.05). Similar to effects of sulfuric acid concentration, lignin removal decreased while crystallinity increased with increasing digestion time. The 15 min digestion time resulted in the highest percentage of lignin removal and lowest crystallinity compared to 45, 60, and 90 min digestion time (P<0.05).

Effect of digestion temperature change on lignocellulosic components and crystallinity is shown in Table 7. Cellulose and hemicellulose hydrolysis increased with increased digestion temperature, lignin removal was decreased, and the crystallinity increased. When digestion temperature was at 80°C, lignin removal was generally larger and the crystallinity generally lesser than other digestion temperatures. However, the hydrolysis of cellulose and hemicellulose was very low. When digestion temperature was increased to 100°C, hemicellulose hydrolysis increased by about 48% compared to 80°C, which suggested that digestion temperature was the primary factor of lignocellulose deconstruction and change of crystallinity.

#### Effect of single factor pretreatments on the concentration of glucose and xylose

**Figure 2:**
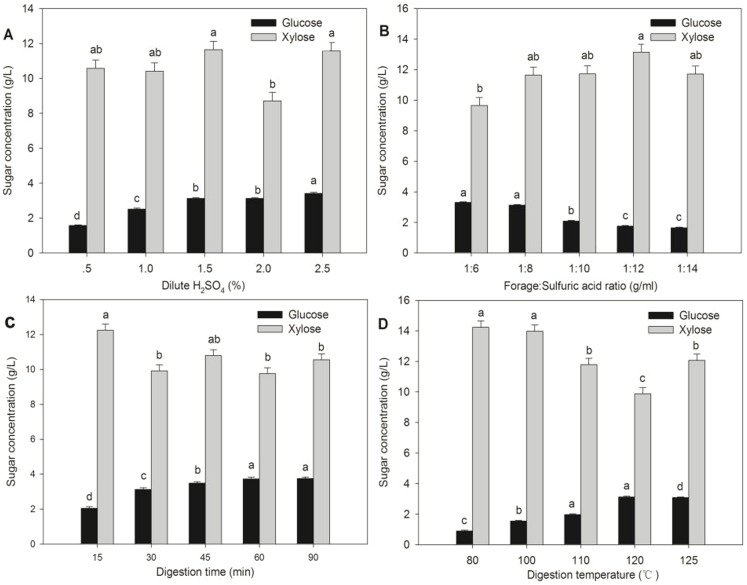
Effect of dilute H_2_SO_4_ pretreatment on sugar concentration at (A) different H_2_SO_4_ concentrations with fixed forage:sulfuric acid ratio (1∶8), fixed digestion time (30 min), and fixed digestion temperature (120°C); (B) different forage:sulfuric acid ratios with fixed H_2_SO_4_ concentration (1.5%), fixed digestion time (30 min), and fixed digestion temperature (120°C); (C) different digestion times with fixed H_2_SO_4_ concentration (1.5%), fix forage:sulfuric acid ration (1∶8), and fixed digestion temperature (120°C); and (D) different digestion temperatures with fixed H_2_SO_4_ concentration (1.5%), fixed forage:sulfuric acid ratio (1∶8), and fixed digestion time (30 min) for *Triarrhena sacchariflora* (Maxim.) Nakai. Analyses were performed in triplicate with the error bars representing the corresponding standard errors.

Changes of the concentration of glucose and xylose post-pretreatment are shown in Fig. 2 for single factor pretreatments of sulfuric acid concentration, forage:sulfuric acid ratio, digestion time, and digestion temperature. The levels of sulfuric acid pretreatment influenced the concentration of glucose and xylose, and xylose concentrations were higher than glucose concentrations with sulfuric acid concentration, forage:sulfuric acid ratio, digestion time, and digestion temperature treatments (P<0.01).

For *Triarrhena sacchariflora* (Maxim.) Nakai, the main hydrolysis products of cellulose and hemicellulose were glucose and xylose after dilute sulfuric acid pretreatment, respectively. In theory, cellulose hydrolysis per 162 kg of cellulose can yield 180 kg glucose, according to the reaction equation: (C_6_H_10_O_5_)_n_+nH_2_O→nC_6_H_12_O_6_; while hemicellulose hydrolysis per 132 kg of hemicellulose can yield 150 kg xylose, according to the reaction equation: (C_5_H_8_O_4_)_m_+mH_2_O→mC_5_H_10_O_5_ [24]. Consequently, sulfuric acid pretreatment appears to be more effective in the hydrolysis of hemicelluloses to produce xylose than hydrolysis of cellulose to produce glucose [25].

Generally, glucose concentration increased with increasing H_2_SO_4_ concentration, forage:sulfuric acid ratio, digestion time, and digestion temperature. There were no clear trends of responses to sulfuric acid concentration or digestion time pretreatments for xylose production. There appeared to be a trend for xylose production to decrease with increasing digestion temperatures, perhaps due to degradation of xylose to furfural at higher temperatures. There was also a general curvilinear increase in xylose with lesser forage:sulfuric acid ratio with around 1∶12 indicating a possible maxima. Again, perhaps xylose was further degraded at greater forage:sulfuric acid ratios and the degradation may have decreased with decreasing forage:sulfuric acid ratios.

### Incomplete factorial experiment

#### Incomplete factorial experimental results and extreme difference analysis

In terms of total sugar concentration, the order of impact of the single factors investigated is shown in table 8: digestion temperature (D)> digestion time (C)> sulfuric acid concentration (A)> forage:sulfuric acid ratio (B), with the optimal level combination of A2B1C1D1. The order of impact of factors for percentage lignin removal was: digestion temperature > sulfuric acid concentration > digestion time > forage:sulfuric acid ratio with the optimal level combination of A2B2C2D2. According to the comprehensive balance method and single factor experiment analysis, A2B1C1D2 treatment combination required less liquid, and had shorter digestion time, greater hemicellulose hydrolysis, and improved cellulose hydrolysis compared to other combinations. Consequently, A2B1C1D2 was designated as the optimal combination of factors, and the best pretreatment conditions were as follows: 1.5% of sulfuric acid concentration, 1∶6 of forage:sulfuric acid ratio, 15 min of digestion time, and 120°C of digestion temperature.

**Table 8:** Results of the incomplete factorial experiment evaluating dilute H_2_SO_4_ pretreatment for *Triarrhena sacchariflora* (Maxim.) Nakai.

Experiment No.		Factors				Total sugar (g/L)	Hemicelluloses hydrolysis (%)
		Sulfuric acid concentration (A)	Forage:sulfuric acid ratio (B)	Digestion time (C)	Digestion temperature (D)		
1		1 (1.0%)	1 (1∶6)	1 (15 min)	1 (110°C)	17.35	34.50
2		1	1	1	2 (120°C)	14.74	65.44
3		1	2 (1∶8)	2 (30 min)	1	15.65	45.73
4		1	2	2	2	12.20	72.00
5		2 (1.5%)	1	2	1	16.30	60.69
6		2	1	2	2	12.59	82.38
7		2	2	1	1	17.48	53.37
8		2	2	1	2	14.35	77.08
Total sugar	K1	59.94	60.98	63.92	66.78	∑ = 120.66	
	K2	61.72	59.68	56.74	53.88		
	-K1	14.99	15.25	15.98	16.70		
	-K2	15.43	14.92	14.19	13.47		
R		0.44	0.33	1.79	3.23		
Hemicelluloses hydrolysis	K1	217.67	243.01	230.39	194.29	∑ = 491.19	
	K2	273.52	248.18	260.80	296.90		
	-K1	54.42	60.75	57.60	48.57		
	-K2	68.38	62.05	65.20	74.23		
R		13.96	1.30	7.60	25.66		

K1 = Sum of index value of factor1, K2 =  Sum of index value of factor2, K1-bar = K1/2, K2-bar = K2/2.

#### Enzymatic hydrolysis and fermentation

**Figure 3:**
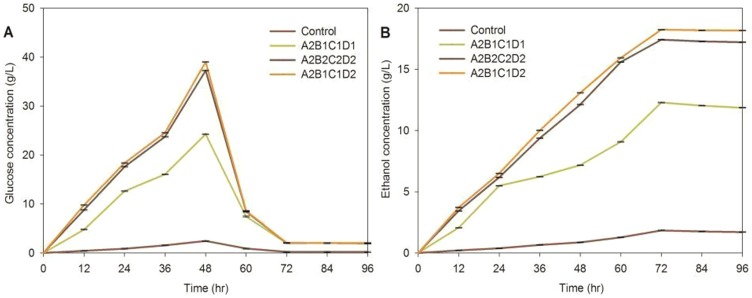
Sugar and ethanol concentrations for 96 h of enzymatic and fermentation for *Triarrhena sacchariflora* (Maxim.) Nakai pretreated at untreated, A2B1C1D1 (1.5%, 1∶6, 15 min, 110°C), A2B2C2D2 (1.5%, 1∶8, 30 min, 120°C), A2B1C1D2 (1.5%, 1∶6, 15 min, 120°C). Analyses were performed in triplicate with the error bars representing the corresponding standard errors.

Glucose and ethanol yields for four optimal pretreatment process conditions are shown Fig. 3. The highest value of glucose was at 48 h digestion, while that of ethanol was at 72 h digestion. Glucose and ethanol concentrations of the A2B1C1D2 process were greatest, slightly greater than those of A2B2C2D2 pretreatment process (P>0.05), and greater than A2B1C1D1 and the control (P<0.05). Predictably, glucose and ethanol concentrations of untreated *Triarrhena sacchariflora* (Maxim.) Nakai were very low. Therefore, pretreatment is critical for improvement in enzymatic hydrolysis of cellulose and hemicellulose. Under the conditions of the A2B1C1D2 pretreatment process, 80% of the cellulose was hydrolysed in 96 h, and 78% converted to ethanol.

## Discussion

The single factor experiments showed that digestion temperature had the greatest effect on the dry matter digestion and there were very large differences among the different digestion temperature treatments (P<0.01). The analyses of sugar concentration indicated that xylose concentration was significantly higher than glucose concentration for most of the different treatments, which showed that dilute sulfuric acid pretreatment was effective in hydrolysing hemicellulose into xylose, but less effective in hydrolysing cellulose to glucose, similar to other research results [26]. Consistent with the objective of breaking down hemicellulose and leaving cellulose for subsequent enzymatic hydrolysis [27], sulfuric acid pretreatment appears to be useful in increasing the efficiency of glucose production and thus ethanol from forages.

Even though hemicelluloses hydrolysis increased with increasing digestion time and temperature, xylose concentration decreased with greater digestion time and temperature. It seems reasonable to hypothesize that the increasing digestion time and temperature facilitated the degradation of xylose to furfural, acetic acid, and phenol, which could inhibit fermentation, but the inhibitors levels of furfural, HMF, acetic acid and formic acid after dilute sulfuric acid pretreatment were very low, which were 1.00–1.37 g/L, undetected, 1.80–2.60 g/L, and 0–0.60 g/L, respectively. It is also possible that the increased digestion times and temperatures favored the generation of other five carbon sugars, such as arabinose or xylulose. Results from single factor pretreatments on crystallinity showed that there was a trend for increased crystallinity with increasing in sulfuric acid concentration, and greater digestion times and temperatures, while the change of forage:sulfuric acid ratio basically had no effect on crystallinity. When pretreatment sulfuric acid concentration, digestion time and temperature were less than 1.5%, 30 min and 100°C, respectively, the crystallinity decreased, and was lower than untreated *Triarrhena sacchariflora* (Maxim.) Nakai. This was probably due to greater digestion temperatures and times changing the polymerization mode of cellulose molecules, promoting the hydrolysis of hemicellulose, and making cellulose hydrolyse more easily. Results indicated that crystallinity had an inverse relationship to lignin removal and a proportional relation with hemicellulose hydrolysis. The increased crystallinity likely made structural changes between lignin and carbohydrates, leading to increased difficulty in lignin removal. However, the cellulose accessibility and hemicellulose hydrolysis were not decreased due to the decreased lignin removal. The results of this experiment suggest that hemicelluloses might be the main factor that effects effective deconstruction of forage of low lignin content, instead of lignin.

## Conclusions

Dilute sulfuric acid pretreatment can remove lignin, hydrolyse a major proportion of hemicelluloses, improve the enzymatic hydrolysis of cellulose, and reduce the crystallinity. However, the results of this experiment suggest that hemicelluloses are the key deconstruction factor in plants with low lignin content such as *Triarrhena sacchariflora* (Maxim.) Nakai. The factors associated with the effect of dilute sulfuric acid pretreatment on ethanol production from *Triarrhena sacchariflora* (Maxim.) Nakai were in order of importance: digestion temperature > sulfuric acid concentration > digestion time > forage:sulfuric acid ratio. The optimum process conditions were: sulfuric acid concentration of 1.5%, forage:sulfuric acid ratio of 1∶6, the digestion time of 15 min, and the digestion temperature of 120°C. Under the conditions of this process, cellulose enzymatic hydrolysis and conversion of ethanol were 80% and 78%, respectively.
